# Introduction to Editorial Board Member: Professor Antonios (Tony) G. Mikos

**DOI:** 10.1002/btm2.10155

**Published:** 2020-01-06

**Authors:** Vassilios I. Sikavitsas

**Affiliations:** ^1^ School of Chemical, Biological, and Materials Engineering The University of Oklahoma Norman Oklahama

In this issue of *Bioengineering and Translational Medicine*, I am excited to introduce the Editorial Board Member, Prof. Antonios (Tony) G. Mikos. Prof. Mikos is the Louis Calder Professor of Bioengineering, Chemical and Biomolecular Engineering, and Director of the National Institutes of Health Center for Engineering Complex Tissues, the Center for Excellence in Tissue Engineering, and the J. W. Cox Laboratory for Biomedical Engineering at Rice University. He received his diploma (1983) in Chemical Engineering from the Aristotle University of Thessaloniki, Greece, and his M.S. (1985) and Ph.D. (1988) in Chemical Engineering from Purdue University under the direction of Prof. Nicholas A. Peppas. He did postdoctoral studies at the Massachusetts Institute of Technology and the Harvard Medical School working with Prof. Robert Langer and Joseph Vacanti before joining Rice University in 1992.

Prof. Mikos is a recognized world leader in the areas of biomaterials, tissue engineering, and controlled release. His research has been extremely influential especially in the synthesis, processing, and evaluation of new orthopedic, dental, cardiovascular, neurologic, and ophthalmologic biomaterials to be applied in (a) tissue engineering strategies, (b) controlled release of drugs, (c) nonviral vectors for gene therapy, and (d) the development of environments to model diseases including cancer.

Prof. Mikos' pioneering research in the development of biodegradable polymeric scaffolds for bone regeneration focused on the development of cell/scaffold constructs that can promote bone formation in vivo. To accomplish this goal, he has employed the use of flow perfusion bioreactors that minimized the nutrient gradients to the cultured pre‐osteoblastic cells residing deep inside porous scaffolds (based on poly(l‐lactic‐*co*‐glycolic) acid, poly(l‐lactic) acid, poly(ε‐caprolactone), titanium, and starch among others) while at the same time stimulating the osteoblastic differentiation of these cells utilizing their exposure to shear forces generated by the media perfusion.^1^, ^2^ He also demonstrated the osteoinductive potential of the in vitro generated extracellular matrix (ECM) by the cultured cells after decellularizing the precultured cell/scaffold constructs and reseeding them with new cells on the remaining ECM.^3^

With the advancement of rapid prototyping technologies, Prof. Mikos targeted the creation of uniform and gradient scaffold architectures for bone and osteochondral defect repair. The incorporation of hydroxyapatite nanoparticle gradients in polypropylene fumarate or poly(ε‐caprolactone) scaffolds has the potential to recapitulate gradients found in native osteochondral tissues.^4^ His approach attempts to mimic the hierarchical features of native tissue specific matrix, ranging from molecular composition to nano/microscale biochemical and physical features. Nanocomposites using nanoparticles and single‐walled carbon nanotubes as reinforcing agents to improve mechanical properties of bone tissue engineering scaffolds have also been explored in Prof. Mikos' laboratory.^5^

Beyond the use of precultured cell/scaffold constructs for orthopedic applications, Prof. Mikos worked intensely toward the fabrication of injectable, in situ polymerizable, biodegradable composite scaffolds that can be incorporated in damaged bone, cartilage, or even cardiac tissue. His work in the development of injectable polymers involved, among other polymers, the use of oligo(poly[ethylene glycol] fumarate),^6^ poly(propylene fumarate‐*co*‐ethylene glycol),^7^ and copolymer macromers of *N*‐isopropylacrylamide.^8^ The flexibility of thermally and chemically gelating polymer systems in orthopedic surgery, which allows the delivery of cells, growth factors, or even genes, in irregular defect sites, attracted his attention.

The delivery of biologic components from tissue engineering scaffolds has been central in the strategies explored by Prof. Mikos through the years. The release of bone morphogenetic proteins (BMP2 and BMP7) together with the release of transforming growth factor, β3, from hydrogels and prefabricated solid porous polymeric scaffolds are examples of his efforts to develop methodologies for the enhancement of osteogenesis and chondrogenesis.^9^ He also managed to successfully transfer genes to cells using poly(ethylenimine)/DNA complexes.^10^

Prof. Mikos utilized many of his advancements in the areas of biomaterials and tissue engineering in the development of three‐dimensional (3D) in vitro cancer models. He established an ex vivo 3D Ewing sarcoma model that mimicked the morphology, growth kinetics, and protein expression profile of human tumors, overcoming the limitations of two‐dimensional cancer cell culture systems. He incorporated flow perfusion in the 3D Ewing sarcoma model incorporating the biomechanical stimulatory effect of fluid shear on the cultured tumor cells that drives tumor progression.^11^ He has also utilized his expertise in creating scaffolds with tunable stiffness to elucidate the effect of the substrate stiffness on osteosarcoma, specifically the localization and expression of the mechanoresponsive proteins, YAP and TAZ.^12^

Prof. Mikos is the author of more than 600 publications and the inventor of 29 patents. His papers received more than 70,000 citations and he has an h‐index of 146. He is a member of the National Academy of Engineering, the National Academy of Medicine, the National Academy of Inventors, and the Academy of Athens. He has been recognized by many prestigious awards including the Lifetime Achievement Award of the Tissue Engineering and Regenerative Medicine International Society‐Americas, the Founders Award of the Society for Biomaterials, the Robert A. Pritzker Distinguished Lecturer Award of the Biomedical Engineering Society, and the Marshall R. Urist Award for Excellence in Tissue Regeneration Research of the Orthopedic Research Society. He is a founding editor and editor‐in‐chief of the journal *Tissue Engineering*.

On behalf of his former doctoral and postdoctoral students (more than 70 and 40, respectively) and his current students, I express my gratitude for his dedication to mentoring scientists throughout their careers, and his awe‐inspiring accomplishment is science and engineering.

**Figure 1:**
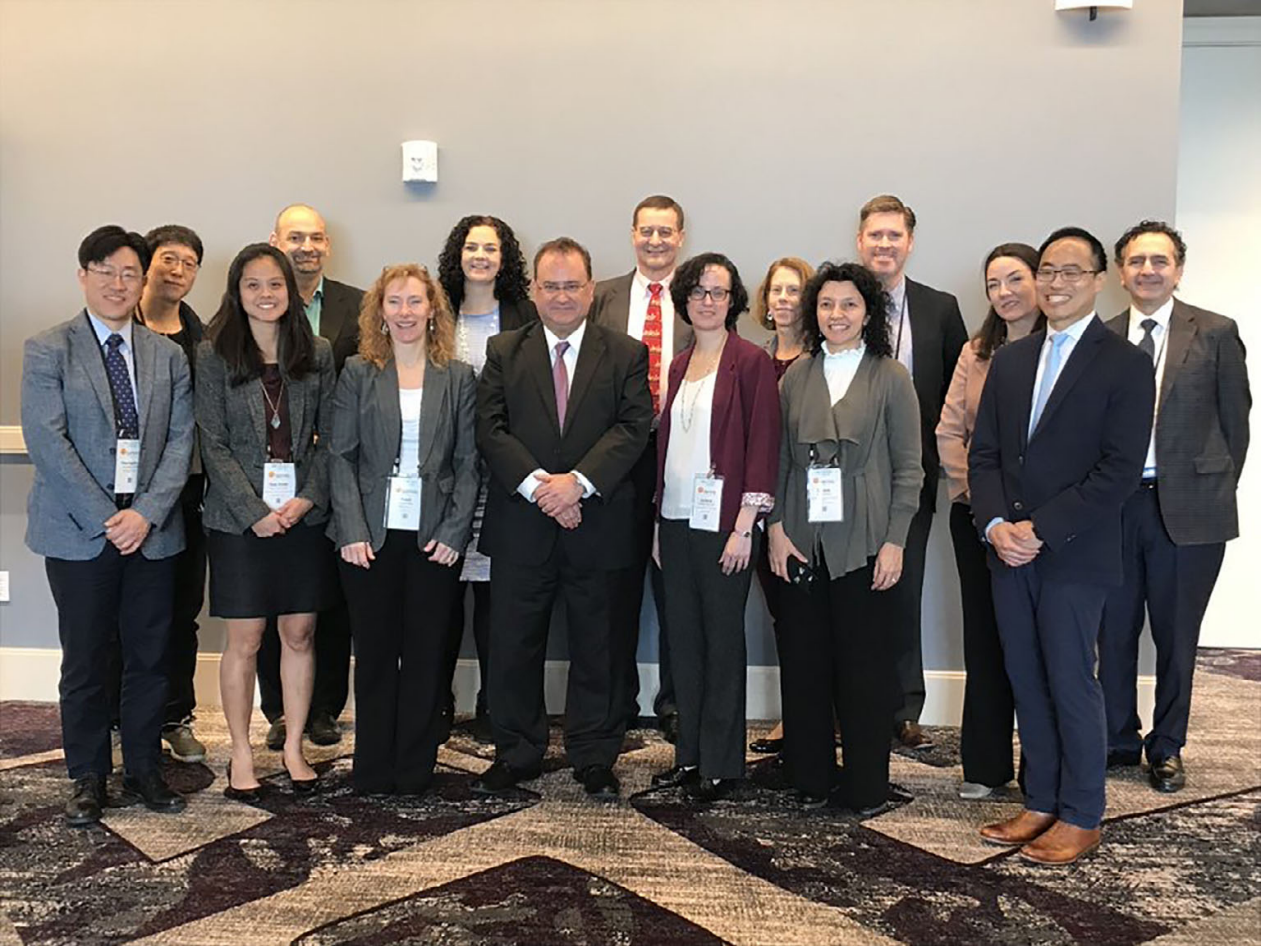
Tony pictured with presenters (previous students and current collaborators) from his 60th birthday Symposium at the TERMIS‐AM 2019 Conference in Orlando, Florida. Photo courtesy of Dr. Elizabeth Cosgriff‐Hernandez
